# Multidimensional risk factor analysis of acute low back pain progressing to chronicity: a longitudinal cohort study protocol

**DOI:** 10.3389/fmed.2023.1194521

**Published:** 2023-06-26

**Authors:** Yilong Huang, Chunli Li, Jiaxin Chen, Yuanming Jiang, Yingjuan Yang, Juntao Yang, Zhongwei Wang, Derong Zhao, Mingbin Luo, Fushun Pu, Zhenguang Zhang, Bo He

**Affiliations:** ^1^Department of Medical Imaging, The First Affiliated Hospital of Kunming Medical University, Kunming, China; ^2^Department of Radiology, Dali Bai Autonomous Prefecture People's Hospital, Dali, China; ^3^Department of Radiology, Baoshan People’s Hospital, Baoshan, China; ^4^Department of Radiology, The First People’s Hospital of Honghe State, Mengzi, China

**Keywords:** acute low back pain, chronicity, risk factors, MRI, protocol

## Abstract

**Introduction:**

Approximately 40% of patients with acute low back pain (LBP) develop chronic low back pain, which significantly increases the risk of poor prognosis. To reduce the risk of acute LBP becoming chronic, effective preventive strategies are needed. Early identification of risk factors for the development of chronic LBP can help clinicians choose appropriate treatment options and improve patient outcomes. However, previous screening tools have not considered medical imaging findings. The aim of this study is to identify factors that can predict the risk of acute LBP becoming chronic based on clinical information, pain and disability assessment, and MRI imaging findings. This protocol describes the methodology and plan for investigating multidimensional risk factors for acute LBP becoming chronic, in order to better understand the development of acute LBP and prevent chronic LBP.

**Methods:**

This is a prospective multicenter study. We plan to recruit 1,000 adult patients with acute low back pain from four centers. In order to select four representative centers, we find the larger hospitals from different regions in Yunnan Province. The study will use a longitudinal cohort design. Patients will undergo baseline assessments upon admission and will be followed up for 5 years to collect the time of chronicity and associated risk factors. Upon admission, patients will be collected detailed demographic information, subjective and objective pain scores, disability scale, and lumbar spine MRI scanning. In addition, patient’s medical history, lifestyle, psychological factors will be collected. Patients will be followed up at 3 months, 6 months, 1 year, 2 years and up for 5 years after admission to collect the time of chronicity and associated factors. Multivariate analysis will be used to explore the multidimensional risk factors affecting the chronicity of acute LBP patients (such as age, gender, BMI, degree of intervertebral disc degeneration, etc.), and survival analysis will be performed to explore the impact of each factor on the time of chronicity.

**Ethics and dissemination:**

The study has been approved by the institutional research ethics committee of each study center (main center number: 2022-L-305). Results will be disseminated through scientific conferences and peer-reviewed publications, as well as meetings with stakeholders.

## Introduction

Low back pain (LBP) is a common health problem and has become a global public health threat (1, 2). LBP is classified as acute LBP, sub-acute LBP and chronic LBP, depending on the duration of the pain (3). There is a close relationship between acute LBP and chronic LBP. Acute LBP is often self-limited and resolves within a few weeks (4), but approximately 40% of patients may develop chronic LBP, Chronic LBP is associated with a poor prognosis (5). To prevent acute LBP from progressing to chronic LBP, effective prevention strategies are needed. One approach to treatment is not effective for all LBP patients (6), and it is essential to identify patients who are at risk of developing chronic LBP.

Previous studies have investigated the risk of acute LBP progressing to chronicity and have shown that numerous factors can affect the recovery process, including biological, psychological, and social factors (7–9). These factors include lack of physical activity, mental health issues, economic pressure, and work environment, among others. Additionally, the severity and duration of acute LBP are related to the risk of chronicity (10). However, currently there are very limited preventive measures for acute LBP developing into chronic LBP (11), which may be related to single-center and single-parameter dimensions.

Imaging are not routinely performed for acute nonspecific LBP (12). However, imaging may be necessary when a patient presents with “red flag” (2) symptoms, suspected structural spine damage, or persistent or recurrent pain (13). Magnetic resonance imaging (MRI) has emerged as a valuable tool for the assessment and diagnosis of acute LBP, even predict disability (14). MRI can provide detailed anatomical images of the spine and detect the presence of disc herniation, nerve root compression, and other structural abnormalities (15, 16). This imaging modality can also provide valuable information on the severity of the injury and guide treatment decisions. The systematic review shows that MRI findings are associated with future pain and functional impairment in patients with acute LBP (14). It should be noted that MRI findings alone cannot fully predict the likelihood of acute LBP progressing to chronic LBP, as this process is complex and may be influenced by biological, psychological, and social factors.

Therefore, the purpose of this study is to identify multidimensional risk factors associated with first-time acute LBP progressing to chronic LBP by using clinical information, pain level, disability assessment, and MRI imaging. The study aims to provide a comprehensive evaluation of the risk factors associated with chronic LBP and identify patients who are at risk of developing chronic LBP.

## Methods and analysis

This multicenter longitudinal cohort study conducted in Yunnan province, China, aims to identify the risk factors associated with the transition of acute LBP to chronic LBP based on multidimensional clinical and MRI medical information. This study could provide personalized treatment strategies for patients with acute LBP and to provide a measure for preventing the development of chronic LBP from acute LBP.

### Study design

**Figure 1:**
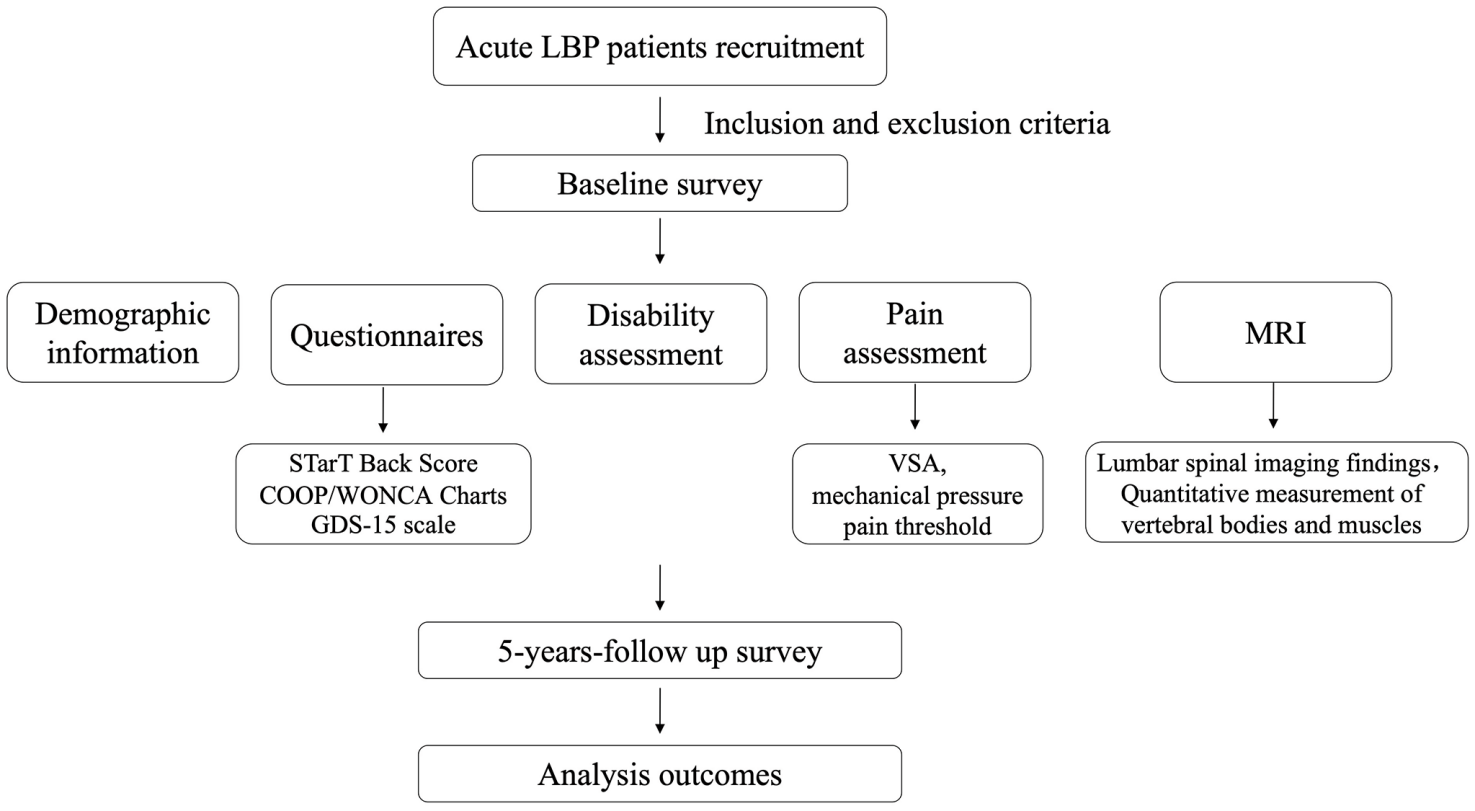
Flow chart of study design and procedures. LBP, low back pain.

This LBP study is a multicenter, prospective, cohort study. The study protocol consists of three main steps: subject recruitment, baseline survey, and 5 years follow-up survey (Figure 1).

### Subject recruitment

The eligibility of potential acute LBP patients is assessed by investigators from each clinical center according to the inclusion and exclusion criteria. Inclusion criteria (1): first-time acute LBP patients with a duration from 24 h to 4 weeks (2); age 18 years or older (3); pain intensity: moderate, items 7 of SF-36, or VAS > 2 (4); preceded by a period of at least 1 month without pain. Exclusion criteria: (1) contraindications for MRI examination or inability to cooperate with scanning; (2) visceral-origin LBP (e.g., urolithiasis); (3) spinal trauma, tumor, infection, surgery, intraspinal tumor; (4) kidney diseases; (5) ankylosing spondylitis; (6) pregnancy status. Recruitment started in January 2022. Each participant will sign an informed consent form before officially entering the study.

Ethical approval for the cohort study is obtained from the ethics committee of each study center [the First Affiliated Hospital of Kunming Medical University, No. 2022-L-305]. The study is conducted by ethical principles according to the Declaration of Helsinki. Informed consent is obtained from each participant at the nearest participating imaging center.

### The estimation of sample size

The current study aims to recruit 1,000 participants. The reported overall prevalence of acute LBP in adults is 80%. The possibility of progression of acute LBP to chronicity is reported to be 40%. Previous study showed a probability of approximately 33% for chronicity within 24 months (17). These probabilities are taken into account in determining the sample size for the study. Thus, the number of 1,000 is set to get the estimated overall prevalence of acute LBP to be within 5% of the prevalence in the real world and considering the attrition rate of about 20%. This number is also needed to detect significant results from the prospective cohort (survival analysis) with a two-tailed level of significance of 5% and statistical power of 80%. Using SPSS 26.0 software (IBM, Armonk, NY, United States) for data statistics. The level of significance desired for this study is a = 0.05, with a power level of b = 0.2.

### Baseline survey

The demographic information, clinical information, pain assessment, disability questionnaire, muscle strength assessment, and imaging assessments (lumbar spine MRI) are scheduled to be conducted on each participant.

The detailed demographic and clinical baseline information included in this protocol consists of age, gender, height, weight, BMI, duration of symptoms, occupation, working schedule, working environment, work posture, health care, days of sick leave income level, education level, smoking history and annual consumption, alcohol consumption history and annual alcohol consumption, long-term use of corticosteroids, positive result of straight leg raising test, and progressive weakness in the lower extremities.

The STarT Back Score is a simple and effective preliminary screening tool for predicting the prognosis of LBP (18). It consists of nine questions covering various aspects, including the patient’s symptoms, functional status, and psychological factors. Based on the score, patients can be divided into three risk levels: low-risk group, medium-risk group, and high-risk group.

The COOP/WONCA Charts assess the overall health status of individuals (19). The COOP/WONCA Charts include six dimensions of health: physical fitness, feelings, daily activities, social activities, pain, and overall health. The scores range from 1 to 5, with lower scores indicating better health status. The Geriatric Depression Scale (GDS-15) measures the severity of depression and anxiety symptoms in adult individuals (20). The GDS-15 scale consists of 15 items and scores range from 0 to 15, with higher scores indicating greater severity of depression and anxiety symptoms.

**Figure 2:**
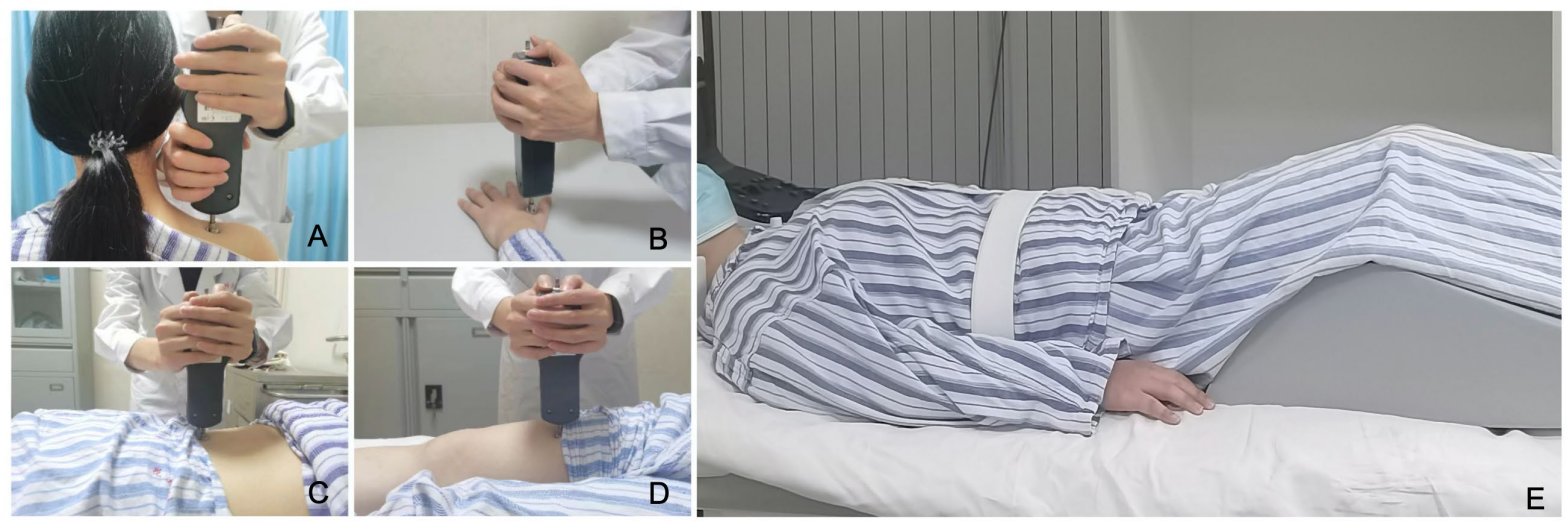
Mechanical pressure pain threshold test and preparing for an MRI examination. **(A–D)** Mechanical pressure gauge be used to measure the pain threshold of patient’s both shoulders, limbs, and back. **(E)** Patient postural fixation before MRI examination.

The degree of pain is assessed using subjective and objective methods. (1) Subjective method: the visual analog scale is used for patients to subjectively rate their pain on a scale of 0–10, where 0 indicates no pain and 10 indicates severe pain that is difficult to tolerate. (2) Objective method: a mechanical pressure gauge is used to objectively measure and record the pain threshold of the shoulders, limbs and paraspinal muscles bilaterally at theL4-5 and L5-S1 levels of the lumbar spine (Figure 2). The measurement is repeated twice with a 10-min interval.

The Oswestry Disability Index (ODI) is a reliable and valid tool for clinical assessment of disability caused by LBP (16). The scale consists of items (pain, lifting, walking, social life, personal care, sitting, standing, sleeping, traveling and sex life), and each scored from 0 to 5. Total ODI score = score of each item × 2, the total ODI score ranges from 0 to 100. A higher total ODI score reflects higher disability. The details of the questionnaires are presented in Table 1.

**Table 1:** The questionnaire in this study.

Questionnaire	Purpose	Number of items	Content
COOP/WONCA Charts	Assess functional status	6	Physical health, mental health, social abilities, occupational abilities, satisfaction with daily activities, and activities of daily living.
GDS-15 scale	Assess depressive symptoms	15	Emotions, vitality, sleep, self-esteem, family, personal health, among others.
STarT Back Score	Assess the risk level of LBP	9	intensity and duration of LBP, the impact of pain on daily activities, anxiety and depressive symptoms, physical activity levels, among others.
VSA	Assess degree of pain	1	A continuous 10 cm line labeled “no pain” and “worst pain” at either end
ODI	Assess degree of disability	10	Ability to perform daily activities, emotional status, social life, pain intensity, among others

GDS-15 scale, 15-item Geriatric Depression Scale; VSA, Visual Analog Scale for Pain; ODI, Oswestry Disability Index; LBP, low back pain.

All clinical centers use a unified equipment of 3.0 T MRI scanner (Discovery 750w, GE Healthcare, USA). The patient’s lumbar spine was fixed and a wedge-shaped foam pad was placed under the lower legs before undergoing MRI (Figure 2). The scanning protocols include conventional sagittal T1-weighted imaging, T2-weighted imaging, and fat-suppressed T2-weighted imaging of lumbar spine, axial T2WI of L1-S1 intervertebral disc, and axial lumbar spine IDEAL-IQ (16). The acquisition coil used is the CTL16-channel spine coil. The MRI scanning parameters are given in Table 2.

**Table 2:** MRI scanning parameters.

Sequences	TR (ms)	TE (ms)	NEX	FOV (cm^2^)	Thickness (mm)	Gaps (mm)	Slices
Sagittal T2WI	2,820	110	3	32 × 32	4	1	15
Sagittal T1WI	378	7.3	2	32 × 32	4	1	15
Sagittal T2 fat-suppressed images	2,500	90	2	32 × 32	4	1	13
Axial T2WI	2,500	90	4	22 × 22	3	0.5	15
Axial IDEAL-IQ	13	5.6	3	24 × 24	4	0	24

T2WI, T2-weighted imaging; T1WI, T1-weighted imaging; IDEAL-IQ, Iterative Decomposition of water and fat with Echo Asymmetry and Least squares estimation - Ideal and Quality; TR, Repetition time; TE, Echo time; NEX, Number of excitations; FOV, Field of view; Gaps, Gap between slices.

Evaluation of morphological features on lumbar spine MRI. Morphological features of lumbar vertebral bodies include: Modic classification (21), degree of vertebral slippage (Meyerding grading) (22), and degree of facet joint degeneration (Weishaupt grading) (23). Morphological features of intervertebral discs include: disc degeneration grade (Pfirrmann grading) (24), type and degree of disc protrusion (MSU classification) (25), degree of spinal canal stenosis (Schizas grading) (26), and degree of neural foramen stenosis (Seunghun grading) (27). The paraspinal muscles are divided into multifidus, and erector spinae, and muscle fat infiltration is assessed using the Goutallier grading system (28).

**Figure 3:**
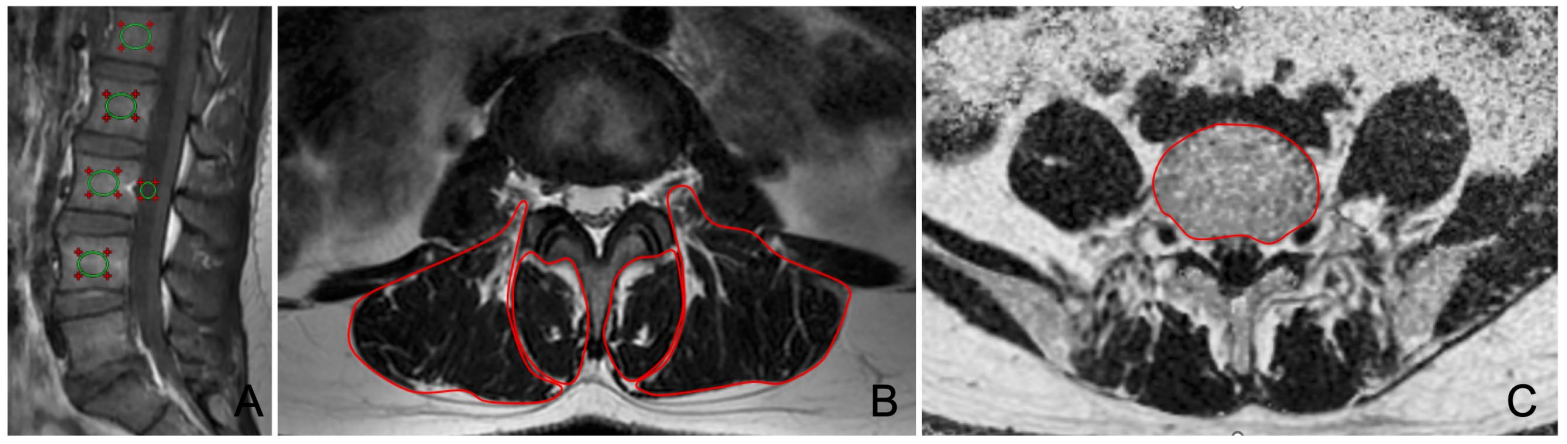
Measurement of VBQ, vertebral body PDFF, paraspinal muscles CSA and PDFF. **(A)** VBQ assessment of vertebral body bone quality on sagittal T1WI image. **(B)** Manual delineation of multifidus and erector spinae on axial T2WI image. **(C)** Manual delineation of vertebral body on axial PDFF map.

Quantitative MRI post-processing and measurement. After completion of MRI scan of the patient, image processing and analysis were performed using a post-processing workstation (Advantage Windows 4.6, GE Medical Systems, USA). Vertebral body quality (VBQ) was measured in the lumbar vertebrae to indirectly evaluate the degree of osteoporosis (29). The calculation formula of VBQ has been extensively described in previous studies, and it is defined as VBQ score = SI_L1-4_/SI_CSF_. Proton density fat fraction (PDFF) obtained with IDEAL-IQ, was used to evaluate the degree of vertebral and paraspinal muscle fat infiltration. The boundaries of the multifidus, erector spinae, and psoas muscles were manually traced, and the cross-sectional area (CSA) of each muscle and the PDFF values of the vertebrae and muscles were recorded. The regions of interest were delineated to avoid tendons and adipose tissue surrounding the muscles. The measurement of VBQ, vertebral body PDFF, paraspinal muscles CSA and PDFF showed in Figure 3.

### Follow-up survey

A 5-year follow-up will be conducted to evaluate the changes in pain level, ODI, MRI findings, muscle CSA, muscle and vertebral PDFF.

### The diagnosis criteria of acute LBP and acute LBP chronicity

LBP is defined as skeletal muscle pain extending from the 12th rib to gluteal fold, sometimes extending into the thigh (above the knee). Depending on the duration of the disease, LBP can be classified as acute (within 6 weeks), subacute (6–12 weeks) and chronic (more than 12 weeks) (30). Chronic LBP are defined as patients experiencing pain in the low back for a duration exceeding 12 weeks, which may be accompanied by sciatica, and cannot be explained by other causes of pain. The chronic status was determined through a questionnaire on chronic LBP. After initial inclusion of patients, we further evaluated the degree of severity of pain and its effect on patients through various measurement tools. This includes Visual Analog Scale (VAS) for measuring the degree of LBP, SF-36 questionnaire evaluation for assessing the impact of pain on life, and recording the frequency and duration of pain. The survey was conducted by researchers collecting data electronically, via email, or over the phone.

### Data management

All data collected will be sent to the First Affiliated Hospital of Kunming Medical University for both analysis and quality control purposes. The data for the study will be gathered and organized in a database constructed with Epidata 3.1. Through this database, all questionnaire contents can be digitized to prepare for further classification, comparison, and statistical analysis, such as clinical information, VSA score, ODI, bone density, muscle PDFF etc. All muscle pain and disability evaluations, MRI scans, and measurements were performed according to the unified standards of the study. The investigators in all centers undergo detailed training before this study begins.

### Statistical analysis

Descriptive statistics were conducted for all variables at baseline and follow-up. At baseline, Pearson’s and Spearman’s rank correlations were computed between variables and VSA and ODI. Independent *t*-tests (continuous variables) and chi-square tests (categorical variables) were used to compare variables between patients with acute LBP without chronicity and those with chronicity. Differences in variables between baseline, 6, 12, and 24 months were compared using one-way ANOVA for continuous variables and chi-square tests for categorical variables. Univariate and multivariate regression analyses were conducted to explore the role of various factors in the transformation from acute to chronic LBP. After the potential predictors are found, test for collinearity between the continuous variables based on Pearson’s *r* > 0.5; multiple logistic regression analyses were conducted using backward stepwise elimination (until none of the variables had value of *p* >0.1) in order to identify the best fitted model with the highest explanatory value (R2); finally, tests for interaction were performed to examine whether any interaction joint increased the model fit, evaluated on Wald tests. Survival analysis methods were used to evaluate the risk of different factors for the transformation from acute to chronic LBP, including Kaplan–Meier survival curves and Cox proportional hazards models. A value of *p* <0.05 was considered statistically significant. During the research process, we planned to delete missing value if less than 5% of predictor values were missing. If more than 5% of predictor values were missing, we planned to impute the missing values through a *post hoc* sensitivity analysis using the Expectation Maximization algorithm in SPSS. Selection bias correction could be accomplished by a generalization of inverse probability weighting estimators. In addition, we attempt to decrease the selection bias by stratification of risk factors.

## Discussion

LBP is a highly prevalent condition in the adult population. Acute LBP carries the risk of progressing to chronic LBP, which has a poorer prognosis. The relationship between acute and chronic LBP is complex, and the risk factors for acute LBP progressing to chronic pain are not fully understood (31). Some biopsychosocial factors have been proposed as potential risk factors, but predicting the chronicity of acute LBP remains challenging (11, 32). It is unclear whether the combination of MRI with clinical information, pain assessment, and disability evaluation is helpful in identifying the risk of acute LBP developing into chronic LBP.

To our knowledge, this is the first study to explore the risk factors for the development of chronicity in acute LBP based on multidimensional medical information. In this five-year longitudinal follow-up cohort study conducted across multiple centers, we observed clinical information, pain levels, disability, detailed MRI features and severity evaluations, as well as changes in bone and muscle fat content of LBP patients during the acute phase. We analyzed the correlation between acute phase clinical information and MRI evaluations with pain and disability at baseline. In longitudinal time, we classified the subjects into two groups based on whether subjects developed chronic LBP, and compared the differences in baseline variables between these two groups. Furthermore, we identified highly correlated factors and risk levels for the chronicity, and evaluated their predictive efficiency.

In conclusion, this study will provide a basis for identifying the multidimensional risk factors for the chronicity of acute LBP, preventing its transition to chronicity, reducing the occurrence of poor prognosis. The results of this study will contribute to the development of effective prevention and personalized treatment strategies for patients with acute LBP.

## Strengths and limitations of this study

The strengths of this study are as follows: Firstly, this is the first multicenter cohort study based on multidimensional medical information to predict the chronicity of acute LBP, effectively controlling confounding factors, and covering risk factors from biological, psychological, sociological, and radiological aspects. Secondly, a variety of indicators, such as detailed MRI features and degree evaluations, changes in bone mass and muscle-fat content, were used to increase the depth and accuracy of the study. This study can provide a deeper understanding of the risk factors for chronicity in patients with acute back pain and provide a basis for early intervention and treatment for patients. In addition, it can provide important clinical guidance and reference for the prevention of chronicity.

This study has potential limitations, as it only observed 5-year longitudinal follow-up data, and longer-term prediction and prognosis assessment may be meaningful. In addition, there may be selection bias in the study population, such as including only LBP patients who seek medical care in hospitals.

## Ethics statement

The studies involving human participants were reviewed and approved by Ethics Committee of the First Affiliated Hospital of Kunming Medical University. The patients/participants provided their written informed consent to participate in this study.

## Author contributions

BH and ZZ: conceptualization, project administration, funding acquisition, supervision, and writing—review and editing. YH and CL: data curation, data analysis, writing—original draft, supervision, and investigators training. YH, CL, JC, YJ, YY, JY, ZW, DZ, ML, and FP: subject recruitment, research data collection, and recording. YH: sample size calculation and statistical analysis. All authors contributed to the article and approved the submitted version.

## Funding

This work was supported by National Natural Science Foundation of China (No. 82260338), Yunnan Provincial Clinical Medical Research Center Project (202102AA100067), the Applied Basic Research Project of Yunnan Province-Kunming Medical University Joint Fund (202201AC070669), and Applied Basic Research Project of Yunnan Province-Youth Project (202201AU070051).

## Conflict of interest

The authors declare that the research was conducted in the absence of any commercial or financial relationships that could be construed as a potential conflict of interest.

## Publisher’s note

All claims expressed in this article are solely those of the authors and do not necessarily represent those of their affiliated organizations, or those of the publisher, the editors and the reviewers. Any product that may be evaluated in this article, or claim that may be made by its manufacturer, is not guaranteed or endorsed by the publisher.
